# Intestinal Epithelial Piezo1–Serotonin Signaling Contributes to Vascular Injury in Steroid-Induced Osteonecrosis of the Femoral Head

**DOI:** 10.3390/ijms27209213

**Published:** 2026-10-16

**Authors:** Lizhi Han, Yuxi Wang, Ping Wang, Yihao Tao, Bo Wang, Xiao Lv, Tao Bai, Yong Feng

**Affiliations:** 1 Department of Orthopedics, Union Hospital, Tongji Medical College, Huazhong University of Science and Technology, Wuhan 430022, China; hlz8566@163.com (L.H.); husterwyx29@163.com (Y.W.); d202582499@hust.edu.cn (P.W.); yihao.tao@outlook.com (Y.T.); wangbob2019@126.com (B.W.); xlyu@hust.edu.cn (X.L.); 2 Division of Gastroenterology, Wuhan Union Hospital, Tongji Medical College, Huazhong University of Science and Technology, Wuhan 430022, China

**Keywords:** Angiogenesis, bone microvascular endothelial cells, Piezo1, serotonin, steroid-induced osteonecrosis of the femoral head, TPH1, type H vessels

## Abstract

Steroid-induced osteonecrosis of the femoral head (SONFH) develops with early microvascular injury, but the systemic signals connecting glucocorticoid exposure to the femoral head remain incompletely defined. We examined whether intestinal epithelial Piezo1–TPH1–serotonin signaling contributes to this process. A glucocorticoid-induced SONFH model was studied in male C57BL/6 mice (8–10 weeks old), using pharmacological inhibition of peripheral serotonin synthesis, intestinal epithelial-specific Piezo1 deletion, micro-computed tomography, histological and vascular analyses, bone microvascular endothelial cell (BMEC) assays, and independent public transcriptomic datasets. Glucocorticoid exposure increased intestinal Piezo1 and TPH1 expression and circulating serotonin, together with loss of CD31^hi^EMCN^hi^ type H vessels and trabecular deterioration. Inhibiting peripheral serotonin synthesis reduced vascular and structural injury. Intestinal epithelial Piezo1 deletion blunted TPH1 upregulation, preserved femoral-head microvasculature, and improved trabecular architecture. In BMECs, serotonin impaired tube formation and increased Bax-, Caspase-3-, and NLRP3-associated signaling; intestinal Piezo1 deletion reduced the corresponding injury signals in vivo. Intestinal epithelial Piezo1–TPH1–serotonin signaling therefore contributes to SONFH-associated microvascular injury in this model.

## 1. Introduction

Steroid-induced osteonecrosis of the femoral head (SONFH) is characterized by progressive destruction of the femoral head and can ultimately lead to joint collapse. Glucocorticoid exposure is a major cause, but the events that connect a systemic steroid challenge to early vascular injury in the femoral head are still incompletely understood. Microvascular impairment appears early in SONFH and precedes advanced structural failure, making the signals that disturb femoral-head endothelial homeostasis particularly relevant to disease initiation [1,2,3]. Osteonecrosis of the femoral head has also been reported in association with antiphospholipid syndrome, in which thrombotic vascular complications may compromise bone perfusion [4].

Peripheral serotonin (5-hydroxytryptamine, 5-HT) provides one possible link between the intestine and the skeleton. Most circulating 5-HT is synthesized in the gut and can influence skeletal homeostasis through endocrine signaling [5,6]. Gut-derived serotonin suppresses osteoblast-mediated bone formation, whereas brain-derived serotonin has distinct central actions, so the skeletal effects of 5-HT depend strongly on its source [7,8,9,10]. Whether an abnormal intestinal serotonergic response also contributes to the vascular component of SONFH has not been established.

Piezo1 is a mechanically activated ion channel expressed in the intestinal epithelium, including enteroendocrine cells, and intestinal Piezo1 has been linked to systemic serotonin production [11,12,13,14]. Serotonin, in turn, can alter endothelial homeostasis and modulate inflammatory and apoptotic responses in vascular cells [5,15,16,17,18,19,20]. This led us to ask whether glucocorticoid exposure engages an intestinal Piezo1–TPH1–5-HT response that affects the femoral-head vasculature. We examined the intestinal response to glucocorticoids, tested the effects of peripheral serotonin inhibition and intestinal Piezo1 deletion in vivo, and assessed the direct effects of serotonin on bone microvascular endothelial cells.

The novelty of this study lies not in the previously described connection between intestinal PIEZO1 and peripheral serotonin synthesis, but in examining whether this extra-skeletal endocrine response contributes to the microvascular component of SONFH. We integrate intestinal epithelial-specific Piezo1 deletion and inhibition of peripheral serotonin synthesis with femoral-head vascular phenotyping and BMEC functional assays. These findings identify intestinal serotonergic signaling as a candidate contributor to vascular injury in the combined LPS–glucocorticoid model, without establishing its clinical utility.

If validated in patients, circulating serotonergic changes could be explored as correlates of early vascular injury, and intestine-restricted modulation of PIEZO1–TPH1 signaling may merit investigation as a potential therapeutic approach. Such clinical applications remain speculative; neither diagnostic performance nor therapeutic efficacy has been tested in patients.

## 2. Results

### 2.1. Glucocorticoid Exposure Induces Sustained Increases in Circulating Corticosterone and Serotonin, Accompanied by Femoral Head Trabecular Deterioration

**Figure 1:**
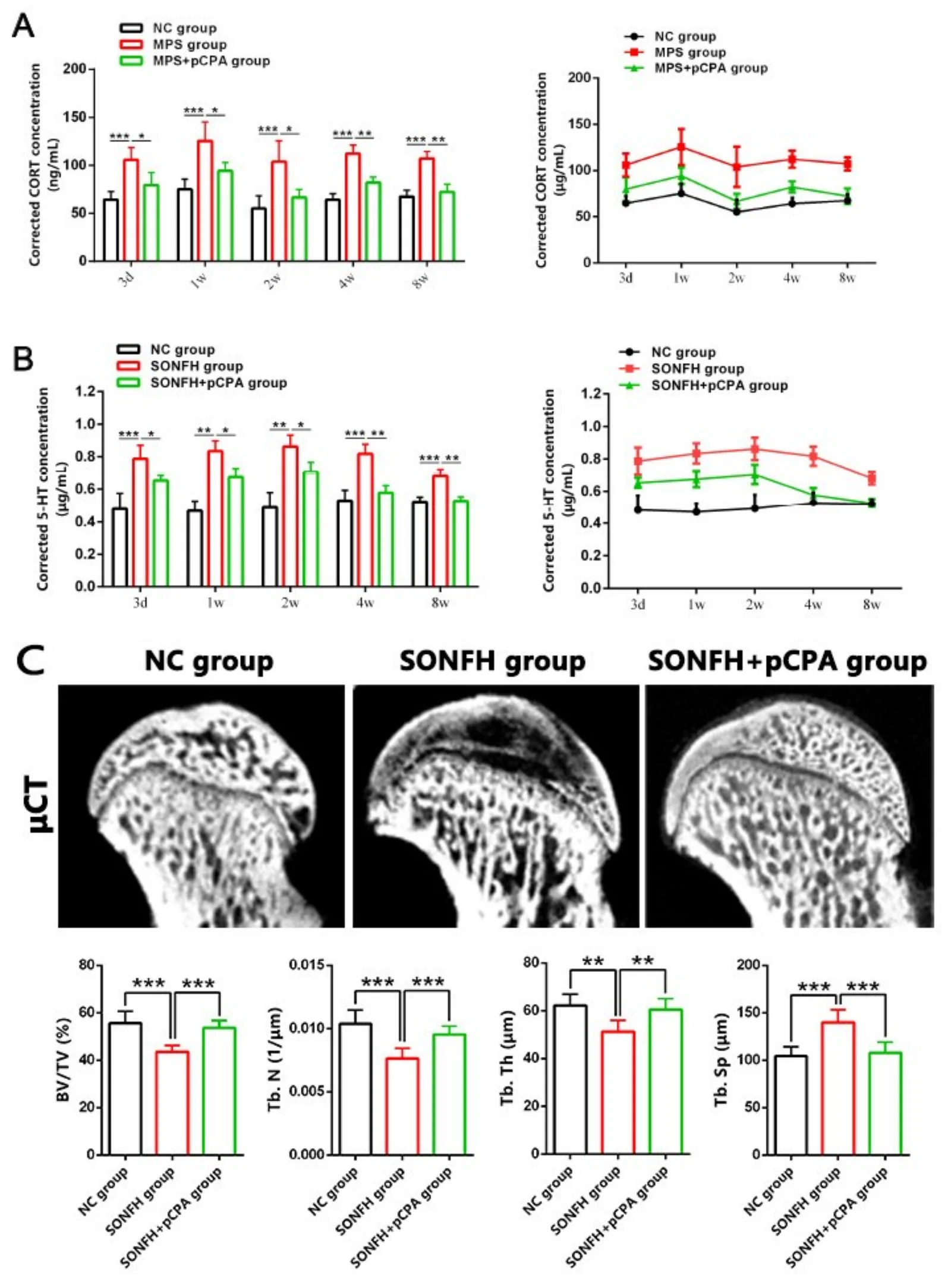
Endocrine changes and trabecular deterioration during SONFH: (**A**) Serum corticosterone (CORT) concentrations in the NC, MPS, and MPS+pCPA groups at 3 days, 1 week, 2 weeks, 4 weeks, and 8 weeks after glucocorticoid administration. Left, bar graph; right, line graph showing dynamic changes over time. (**B**) Serum 5-hydroxytryptamine (5-HT) concentrations in the NC, SONFH, and SONFH+pCPA groups at the indicated time points. Left, bar graph; right, line graph showing dynamic changes over time. (**C**) Representative micro-computed tomography (micro-CT) images of the femoral head in the NC, SONFH, and SONFH+pCPA groups, together with quantitative analysis of bone volume fraction (BV/TV), trabecular number (Tb.N), trabecular thickness (Tb.Th), and trabecular separation (Tb.Sp). Data are presented as mean ± SEM; *n* = 6 mice per group. * *p* < 0.05, ** *p* < 0.01, *** *p* < 0.001. Abbreviations: 5-HT, 5-hydroxytryptamine; BV/TV, bone volume fraction; CORT, corticosterone; micro-CT, micro-computed tomography; MPS, methylprednisolone; NC, normal control; pCPA, p-chlorophenylalanine methyl ester; SEM, standard error of the mean; SONFH, steroid-induced osteonecrosis of the femoral head; Tb.N, trabecular number; Tb.Sp, trabecular separation; Tb.Th, trabecular thickness.

Serum corticosterone (CORT) and serotonin (5-HT) were measured at multiple time points after glucocorticoid administration. CORT was significantly higher in the MPS group than in the NC group from day 3 onward and remained elevated throughout the observation period (Figure 1A). pCPA modestly reduced this increase but did not abolish it (Figure 1A). Serum 5-HT was also higher in SONFH mice and remained elevated from day 3 to week 8 (Figure 1B). pCPA significantly reduced the MPS-induced rise in circulating 5-HT, confirming effective suppression of peripheral serotonin during glucocorticoid exposure (Figure 1B).

To distinguish the contributions of LPS and glucocorticoid exposure to circulating serotonin, an additional four-group comparison (Control, LPS-only, GC-only, and LPS+GC) was performed (Supplementary Figure S3). Relative to controls, neither LPS nor GC exposure alone showed a statistically significant difference in serum 5-HT in the comparisons annotated in the supplied figure. The combined LPS + GC group exhibited higher values than the Control and LPS-only groups, whereas the comparison with GC-only was not significant. These exploratory comparisons do not establish the absence of an LPS contribution or demonstrate a statistically significant interaction between treatments.

Micro-CT showed clear trabecular deterioration in SONFH mice, with lower bone volume fraction (BV/TV), trabecular number (Tb.N), and trabecular thickness (Tb.Th) and higher trabecular separation (Tb.Sp) than in NC mice (Figure 1C). These abnormalities were partially reversed by pCPA (Figure 1C).

### 2.2. Glucocorticoids Upregulate the Intestinal Piezo1–TPH1 Axis Without Altering the CgA-Positive Cell Pool

**Figure 2:**
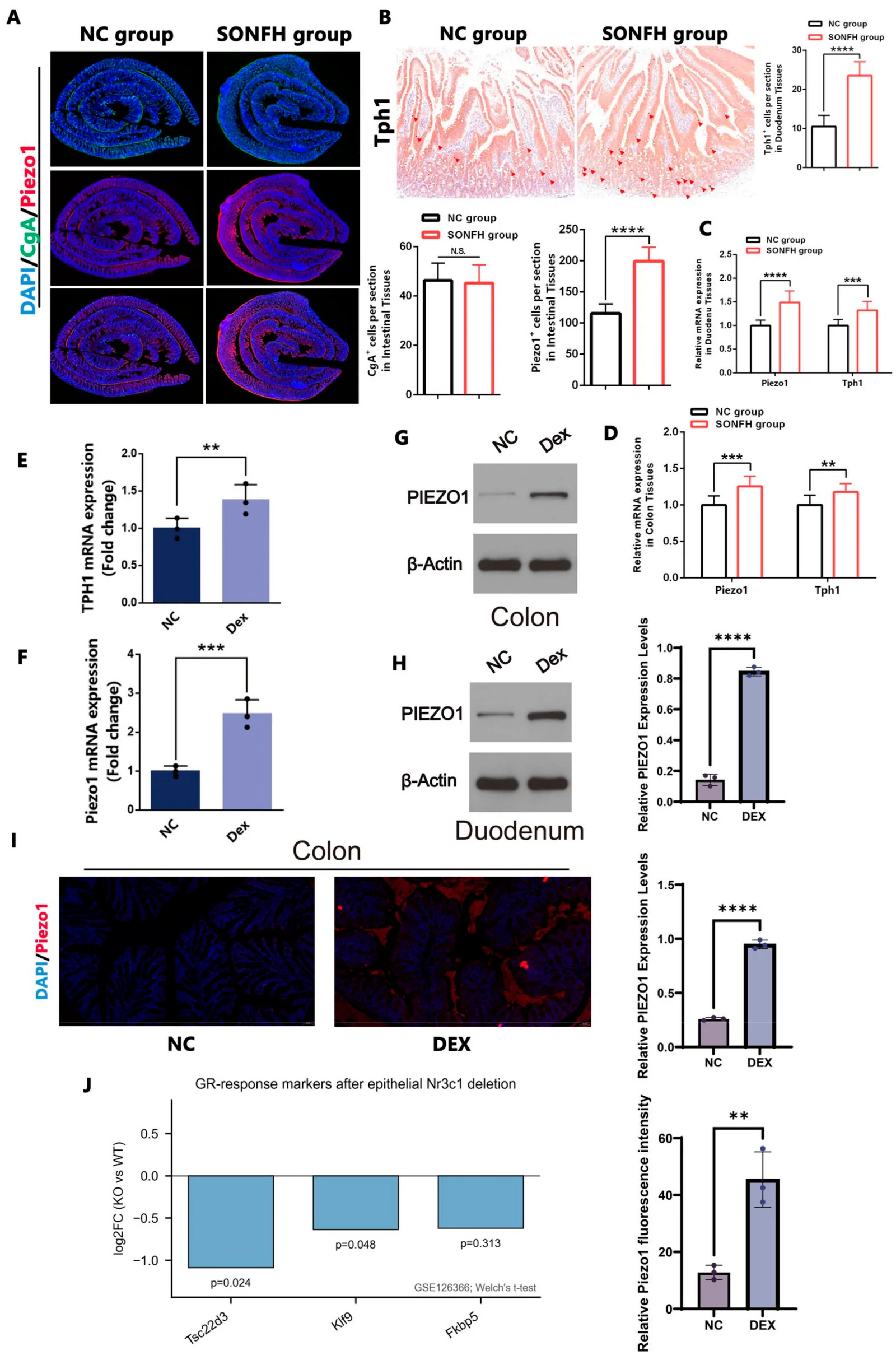
Glucocorticoids upregulate the intestinal Piezo1–TPH1 axis: (**A**) Representative immunofluorescence staining of duodenal sections for Piezo1 and chromogranin A (CgA) in the NC and SONFH groups, with quantification of CgA-positive cells and Piezo1-positive cells per section. Nuclei were counterstained with DAPI. (**B**) Representative immunohistochemical staining of TPH1 in the duodenum from the NC and SONFH groups, with quantification of TPH1-positive cells per section. (**C**,**D**) Relative mRNA expression of Piezo1 and Tph1 in the duodenum (**C**) and colon (**D**) of the NC and SONFH groups, as determined by RT-qPCR. (**E**,**F**) Relative mRNA expression of TPH1 (**E**) and Piezo1 (**F**) in intestinal tissues after dexamethasone (Dex) treatment. (**G**,**H**) Representative Western blot images and quantitative analysis of PIEZO1 protein expression in the colon (**G**) and duodenum (**H**) of NC- and Dex-treated mice. β-Actin was used as the loading control. (**I**) Representative immunofluorescence staining of Piezo1 in the colon from NC- and Dex-treated mice, with quantification of fluorescence intensity. (**J**) GSE126366 intestinal epithelial Nr3c1 perturbation data showing log2 fold changes of GR-response markers after epithelial Nr3c1 deletion. *p* values are from Welch’s *t*-tests. Serotonergic/enteroendocrine gene trends from the same dataset are shown in Figure S1. Data are presented as mean ± SEM. For the Dex-validation RT-qPCR and Western blot panels (**E**–**H**), *n* = 3 biological replicates per group; for the other experimental panels, *n* = 6 mice per group. N.S., not significant; ** *p* < 0.01, *** *p* < 0.001, **** *p* < 0.0001. Abbreviations: CgA, chromogranin A; DAPI, 4′,6-diamidino-2-phenylindole; Dex, dexamethasone; GR, glucocorticoid receptor; mRNA, messenger RNA; NC, normal control; RT-qPCR, reverse-transcription quantitative polymerase chain reaction; SEM, standard error of the mean; SONFH, steroid-induced osteonecrosis of the femoral head; TPH1, tryptophan hydroxylase 1.

The persistent rise in serum 5-HT prompted us to examine the intestinal serotonergic compartment. In duodenal sections, the number of CgA-positive cells was similar in the NC and SONFH groups, whereas Piezo1 immunoreactivity in the intestinal epithelium was markedly increased in SONFH mice (Figure 2A).

TPH1, the rate-limiting enzyme for peripheral serotonin synthesis, was then examined in intestinal tissue. TPH1-positive cells were increased in the duodenum of SONFH mice (Figure 2B), and RT-qPCR showed higher Piezo1 and Tph1 mRNA levels in both the duodenum and colon (Figure 2C,D). Dexamethasone reproduced this response: Tph1 and Piezo1 mRNA increased after Dex treatment (Figure 2E,F), PIEZO1 protein increased in both colon and duodenum (Figure 2G,H), and colon immunofluorescence showed a higher PIEZO1 signal after Dex exposure (Figure 2I).

The unchanged CgA-positive cell pool argues against enteroendocrine-cell expansion as the basis for the increased intestinal PIEZO1 and TPH1 signals.

GSE126366 was examined as an independent intestinal epithelial dataset. After epithelial Nr3c1 deletion, the GR-response markers Tsc22d3 and Klf9 were lower, with Fkbp5 changing in the same direction (Figure 2J). Tph1, Chga, Chgb, Ddc, and Slc6a4 also trended downward, although none of these serotonergic/enteroendocrine genes reached statistical significance (Figure S1).

### 2.3. Pharmacological Inhibition of Peripheral Serotonin Synthesis Preserves Type H Vessels and Attenuates Osteonecrotic Changes in the Femoral Head

**Figure 3:**
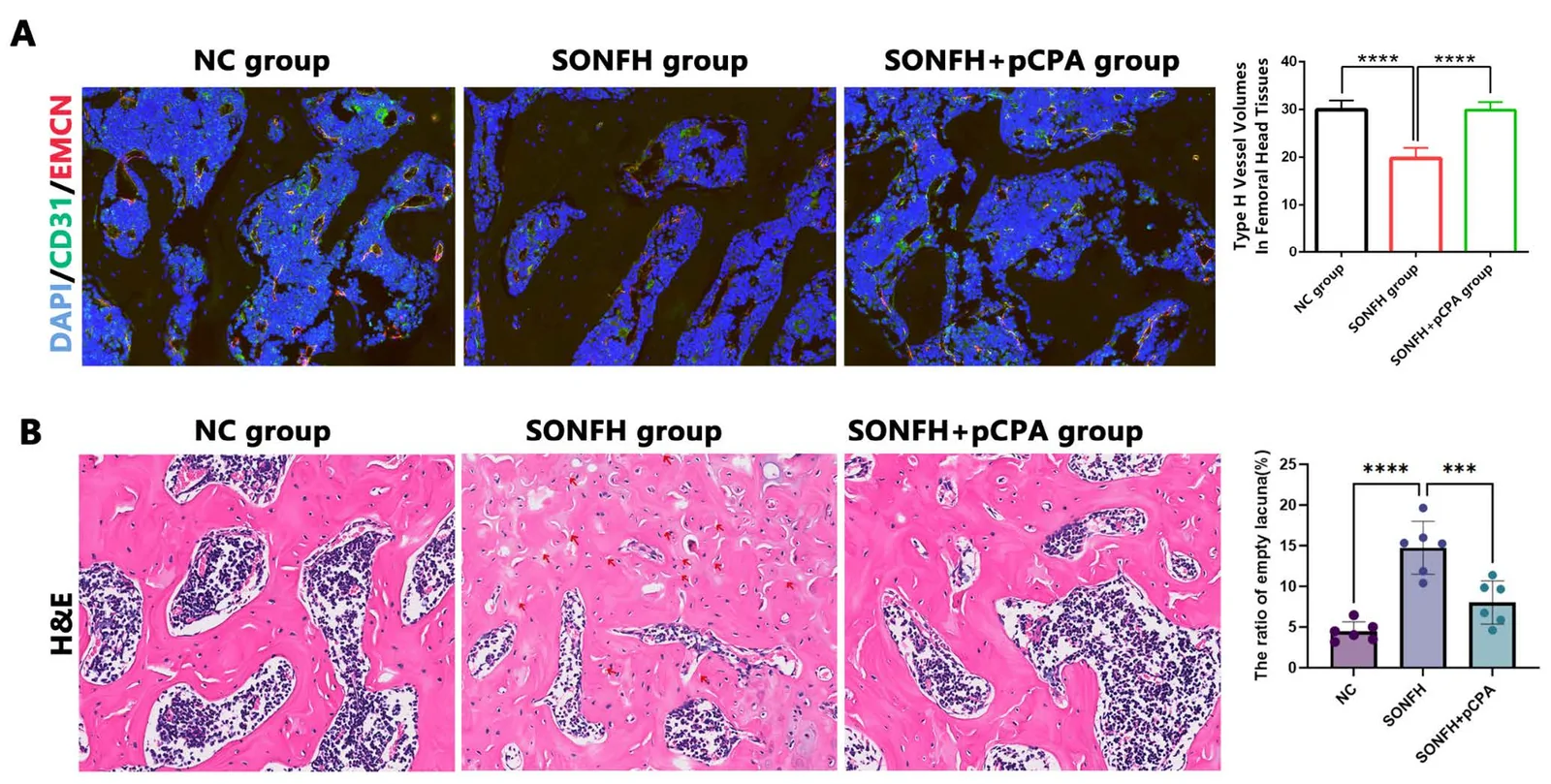
Serotonin inhibition preserves type H vessels in SONFH: (**A**) Representative immunofluorescence staining of femoral head sections for CD31 and endomucin (EMCN) in the NC, SONFH, and SONFH+pCPA groups, together with quantitative analysis of CD31^hi^EMCN^hi^ type H vessel area. Nuclei were counterstained with DAPI. (**B**) Representative hematoxylin and eosin (H&E) staining of femoral head sections in the NC, SONFH, and SONFH+pCPA groups, with quantification of empty osteocyte lacunae as a percentage of total osteocyte lacunae. Red arrows indicate representative empty lacunae. Glucocorticoid treatment induced osteonecrosis-like histological changes, which were alleviated by pCPA administration. Data are presented as mean ± SEM; *n* = 6 mice per group. *** *p* < 0.001, **** *p* < 0.0001. Abbreviations: CD31, cluster of differentiation 31; DAPI, 4′,6-diamidino-2-phenylindole; EMCN, endomucin; H&E, hematoxylin and eosin; NC, normal control; pCPA, p-chlorophenylalanine methyl ester; SEM, standard error of the mean; SONFH, steroid-induced osteonecrosis of the femoral head.

We next asked whether lowering peripheral 5-HT would alter the vascular phenotype in the femoral head. CD31^hi^EMCN^hi^ type H vessel area was markedly reduced in SONFH mice, whereas pCPA treatment restored a substantial proportion of this vascular area (Figure 3A).

H&E staining showed disrupted trabecular architecture, reduced marrow cellularity, and osteonecrosis-like changes in SONFH mice. These changes were less pronounced after pCPA treatment, with better preservation of trabecular structure and less histological evidence of necrosis (Figure 3B). The histological improvement was consistent with the micro-CT findings in Figure 1C. Quantification of H&E sections showed an increased proportion of empty osteocyte lacunae in SONFH mice compared with controls, with a lower proportion following pCPA treatment (Figure 3B).

### 2.4. Intestinal Epithelial Piezo1 Deficiency Suppresses Intestinal Serotonergic Activation and Protects Against Femoral Head Vascular and Osseous Injury

**Figure 4:**
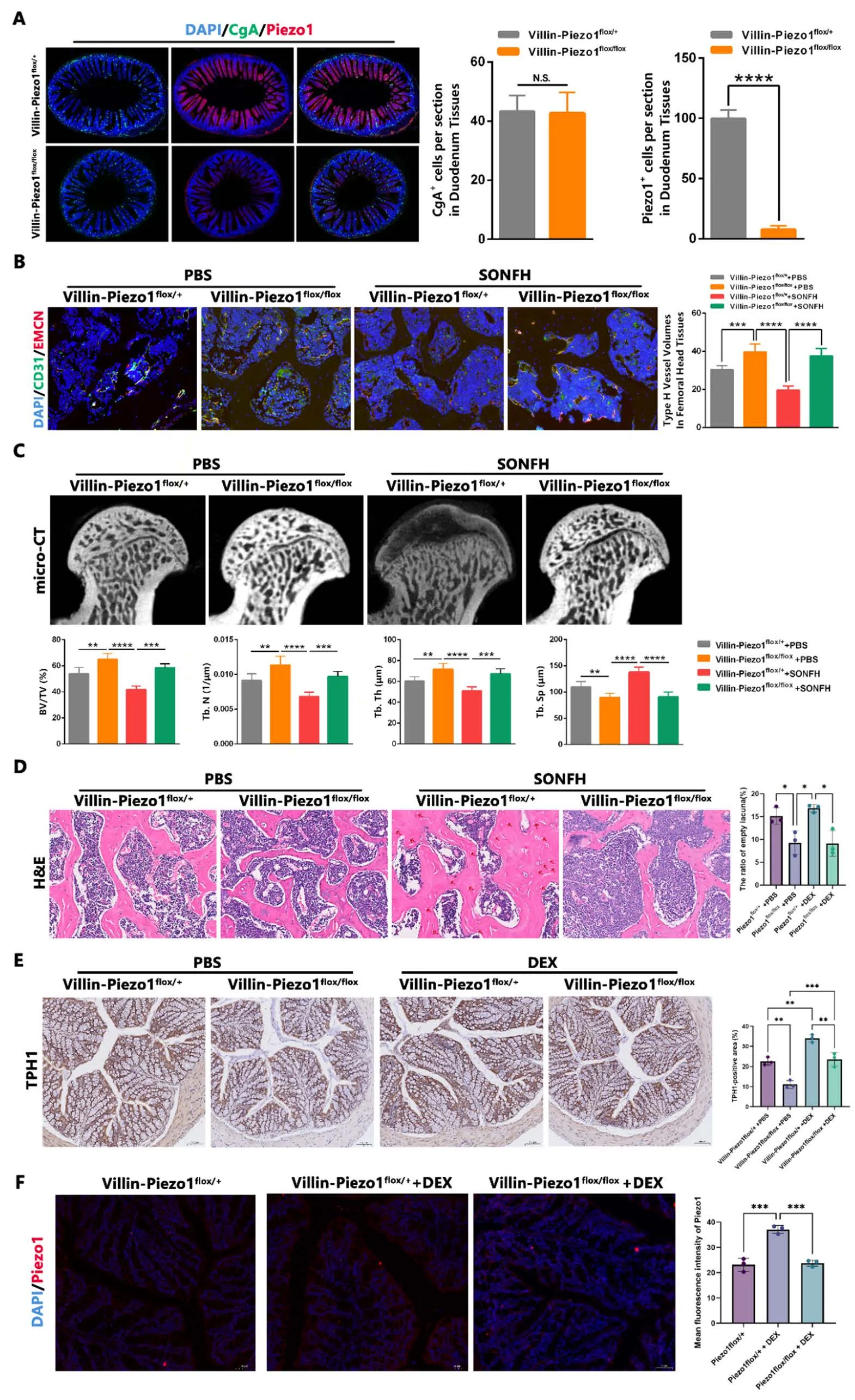
Intestinal Piezo1 deletion protects against femoral head injury: (**A**) Representative immunofluorescence staining of duodenal sections for Piezo1 and CgA in Villin-Piezo1^flox/+^ and Villin-Piezo1^flox/flox^ mice, with quantification of CgA-positive cells and Piezo1-positive cells per section. Nuclei were counterstained with DAPI. (**B**) Representative immunofluorescence staining of femoral head sections for CD31 and EMCN in Villin-Piezo1^flox/+^ and Villin-Piezo1^flox/flox^ mice under PBS or SONFH conditions, with quantification of type H vessel area. (**C**) Representative micro-CT images of the femoral head in Villin-Piezo1^flox/+^ and Villin-Piezo1^flox/flox^ mice under PBS or SONFH conditions, together with quantitative analysis of BV/TV, Tb.N, Tb.Th, and Tb.Sp. (**D**) Representative H&E staining of femoral head sections in the indicated genotype–treatment groups, with quantification of empty osteocyte lacunae as a percentage of total osteocyte lacunae. (**E**) Representative immunohistochemical staining of TPH1 in intestinal tissues from Villin-Piezo1^flox/+^ and Villin-Piezo1^flox/flox^ mice treated with PBS or Dex, with quantification of TPH1-positive area. (**F**) Representative immunofluorescence staining of Piezo1 in intestinal tissues from Villin-Piezo1^flox/+^ mice, Dex-treated Villin-Piezo1^flox/+^ mice, and Dex-treated Villin-Piezo1^flox/flox^ mice, with quantification of Piezo1 fluorescence intensity. Data are presented as mean ± SEM; *n* = 3 mice per group for the empty osteocyte lacunae quantification in panel (**D**), and *n* = 6 mice per group for the remaining panels, unless otherwise stated. N.S., not significant; * *p* < 0.05, ** *p* < 0.01, *** *p* < 0.001, **** *p* < 0.0001. Abbreviations: BV/TV, bone volume fraction; CD31, cluster of differentiation 31; CgA, chromogranin A; DAPI, 4′,6-diamidino-2-phenylindole; Dex, dexamethasone; EMCN, endomucin; H&E, hematoxylin and eosin; micro-CT, micro-computed tomography; PBS, phosphate-buffered saline; SEM, standard error of the mean; SONFH, steroid-induced osteonecrosis of the femoral head; Tb.N, trabecular number; Tb.Sp, trabecular separation; Tb.Th, trabecular thickness; TPH1, tryptophan hydroxylase 1.

To assess the contribution of intestinal Piezo1 to SONFH progression, we generated intestinal epithelial-specific Piezo1 knockout mice. As expected, immunofluorescence staining of duodenal sections showed that the number of CgA-positive cells did not differ between Villin-Piezo1^flox/+^ and Villin-Piezo1^flox/flox^ mice, whereas Piezo1-positive cells were markedly reduced in Villin-Piezo1^flox/flox^ mice, confirming efficient intestinal Piezo1 deletion without altering CgA-positive cell abundance (Figure 4A).

We next assessed whether intestinal Piezo1 deletion affected femoral head vascular injury under SONFH conditions. In PBS-treated mice, the type H vessel area was modestly but significantly increased in Villin-Piezo1^flox/flox^ mice relative to control littermates. After glucocorticoid challenge, type H vessels were markedly reduced in Villin-Piezo1^flox/+^ mice, whereas this reduction was significantly attenuated in Villin-Piezo1^flox/flox^ mice (Figure 4B).

Micro-CT analysis further showed that glucocorticoid-induced bone loss was alleviated by intestinal Piezo1 deletion. Under SONFH conditions, Villin-Piezo1^flox/flox^ mice exhibited higher BV/TV, Tb.N, and Tb.Th and lower Tb.Sp than Villin-Piezo1^flox/+^ littermates (Figure 4C). Consistently, H&E staining revealed better-preserved trabecular architecture and less pronounced osteonecrosis-like changes in Villin-Piezo1^flox/flox^ mice (Figure 4D). Quantitative histological analysis further showed a lower proportion of empty osteocyte lacunae in Villin-Piezo1^flox/flox^ mice than in Villin-Piezo1^flox/+^ controls under both PBS and SONFH conditions (Figure 4D).

To determine whether these protective effects were accompanied by reduced intestinal serotonin synthesis, we examined TPH1 and PIEZO1 expression in intestinal tissues. Dex increased TPH1 expression in Villin-Piezo1^flox/+^ mice, and this increase was attenuated in Villin-Piezo1^flox/flox^ mice (Figure 4E). Intestinal immunofluorescence likewise showed a markedly lower PIEZO1 signal in Dex-treated Villin-Piezo1^flox/flox^ mice than in Dex-treated control mice (Figure 4F). Under glucocorticoid challenge, intestinal Piezo1 deletion was therefore accompanied by reduced TPH1 induction and preservation of femoral-head vasculature and trabecular architecture.

### 2.5. Serotonin Increases NLRP3 and Apoptosis-Related Markers and Impairs BMEC Angiogenic Capacity

**Figure 5:**
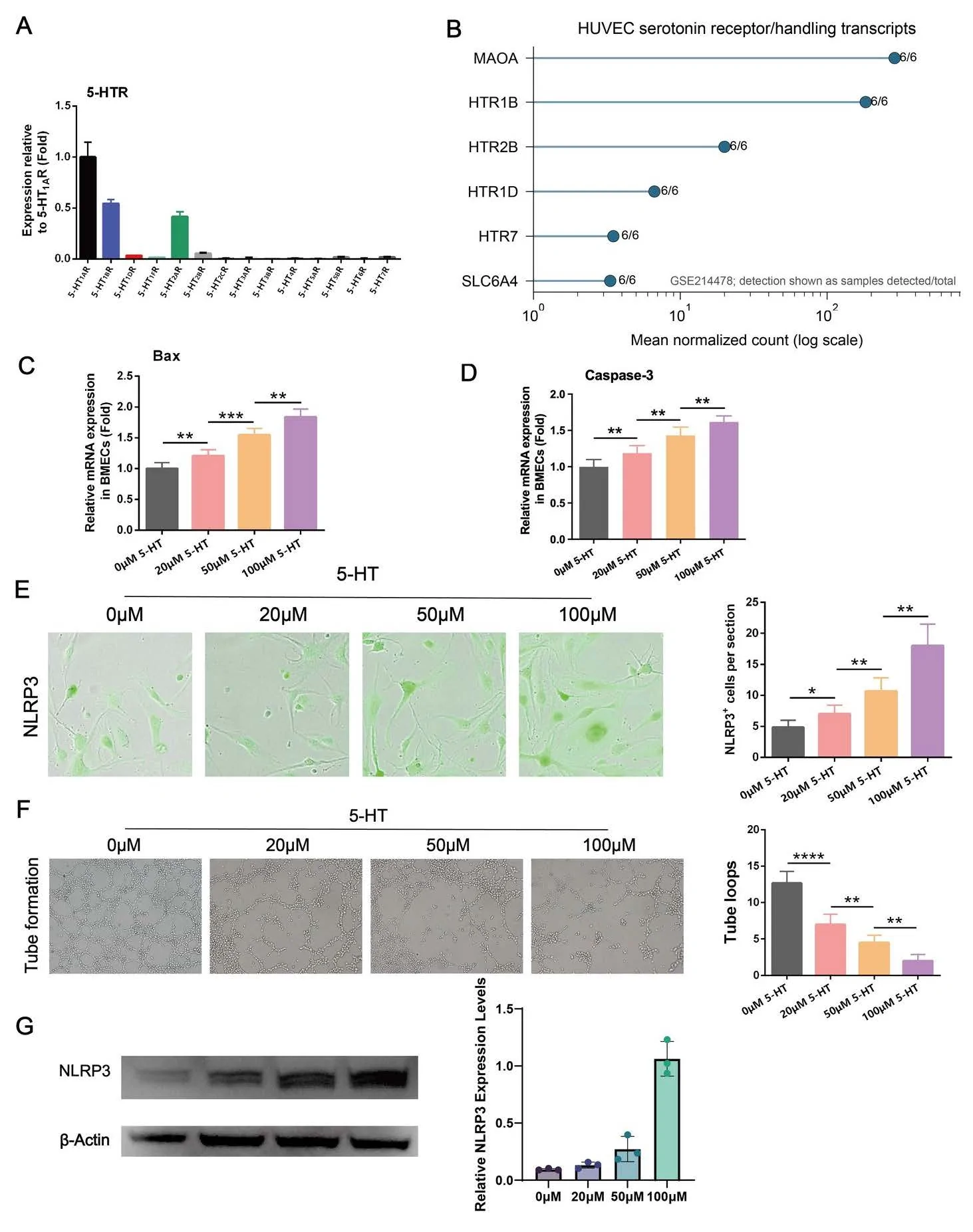
Serotonin impairs BMEC angiogenic capacity and increases NLRP3 and apoptosis-related markers: (**A**) Relative expression of 5-HT receptor family members in bone microvascular endothelial cells (BMECs), normalized to 5-HT1A receptor expression. (**B**) GSE214478 HUVEC RNA-seq data showing detectable serotonin receptor and serotonin-handling transcripts. Detection is shown as samples detected/total, and mean normalized counts are plotted on a log scale. (**C**,**D**) Relative mRNA expression of Bax (**C**) and Caspase-3 (**D**) in BMECs treated with increasing concentrations of 5-HT. (**E**) Representative immunofluorescence staining of NLRP3 in BMECs after treatment with 0, 20, 50, or 100 μM 5-HT, with quantification of NLRP3-positive cells. (**F**) Representative tube formation images of BMECs treated with increasing concentrations of 5-HT, together with quantitative analysis of tube loops. (**G**) Representative Western blot image and quantitative analysis of NLRP3 protein expression in BMECs treated with increasing concentrations of 5-HT. β-Actin was used as the loading control. Data are presented as mean ± SEM. For the NLRP3 Western blot and densitometry in panel (**G**), *n* = 3 independent biological replicates per group; for the other experimental panels, *n* = 6 independent biological replicates per group. * *p* < 0.05, ** *p* < 0.01, *** *p* < 0.001, **** *p* < 0.0001. Abbreviations: 5-HT, 5-hydroxytryptamine; 5-HTR, serotonin receptor; BMEC, bone microvascular endothelial cell; HUVEC, human umbilical vein endothelial cell; mRNA, messenger RNA; NLRP3, NLR family pyrin domain containing 3; RNA-seq, RNA sequencing; SEM, standard error of the mean.

BMECs were exposed to increasing concentrations of 5-HT to determine how serotonin affects bone microvascular endothelial cells directly. Several serotonin receptor subtypes were detected, with relatively higher expression of 5-HT1A, 5-HT1D, and 5-HT2A than of other receptor family members (Figure 5A). GSE214478 HUVEC RNA-seq was examined in parallel and showed detectable HTR1B, HTR1D, HTR2B, HTR7, SLC6A4, and MAOA transcripts (Figure 5B).

Bax and Caspase-3 mRNA increased with rising 5-HT concentrations (Figure 5C,D), while the number of NLRP3-positive BMECs increased in parallel (Figure 5E). Western blotting showed the same concentration-dependent increase in NLRP3 protein (Figure 5G).

The same concentration range impaired BMEC tube formation. Capillary-like networks became progressively less developed as 5-HT increased, and the number of tube loops fell significantly (Figure 5F).

### 2.6. Intestinal Piezo1 Deletion Reduces Dex-Induced NLRP3, Caspase-3, and Bax Signals in the Femoral Head

**Figure 6:**
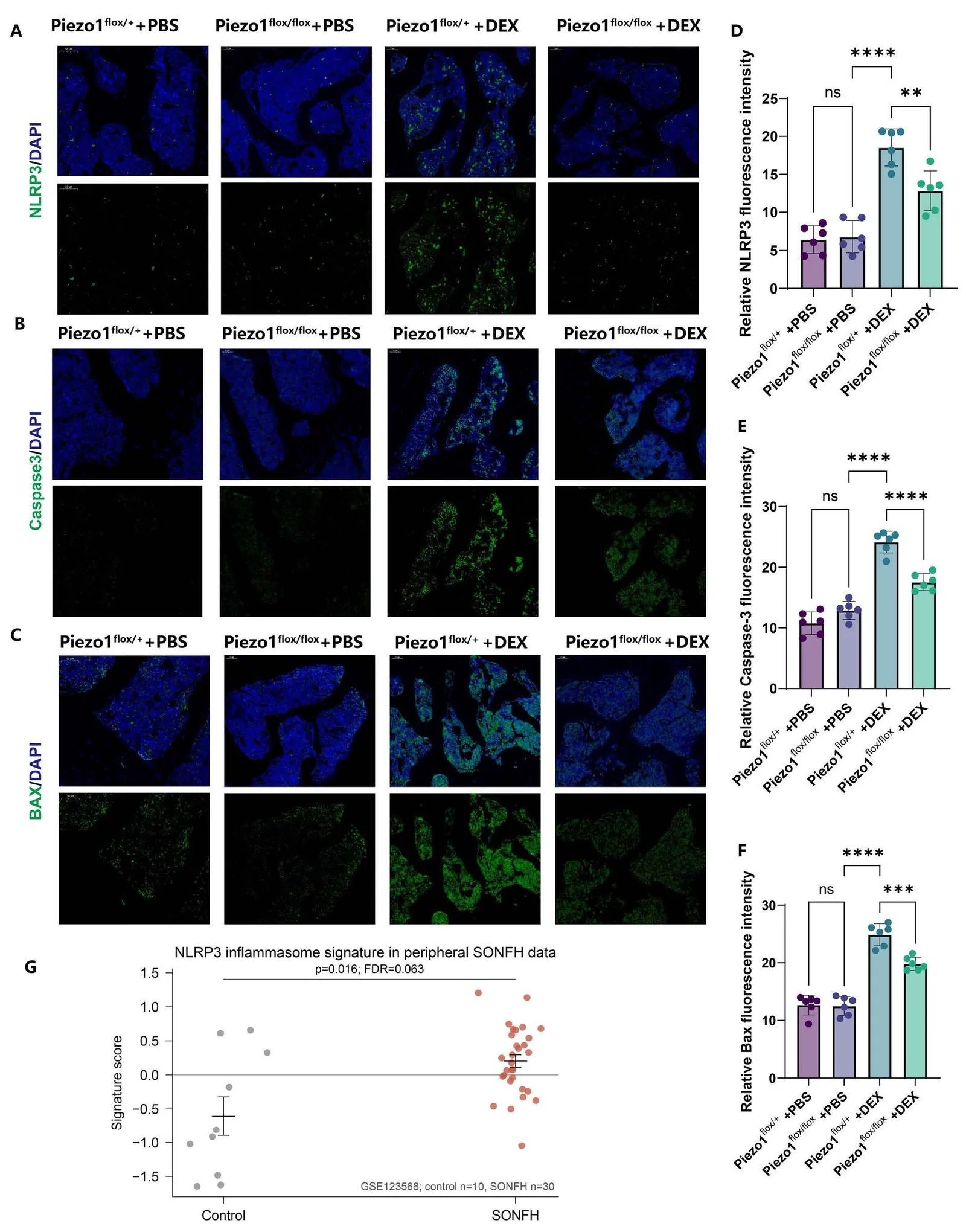
Intestinal Piezo1 deletion reduces Dex-induced NLRP3, Caspase-3, and Bax signals in the femoral head: (**A**–**C**) Representative immunofluorescence staining of NLRP3 (**A**), Caspase-3 (**B**), and Bax (**C**) in femoral head sections from Piezo1^flox/+^ + PBS, Piezo1^flox/flox^ + PBS, Piezo1^flox/+^ + DEX, and Piezo1^flox/flox^ + DEX mice. Upper rows show merged target marker/DAPI images, and lower rows show the corresponding target marker channels. Nuclei were counterstained with DAPI. (**D**–**F**) Relative fluorescence intensity of NLRP3 (**D**), Caspase-3 (**E**), and Bax (**F**) in the indicated groups. (**G**) NLRP3-associated inflammatory signature scores in GSE123568 peripheral SONFH and control samples. Data are shown as individual samples with mean ± SEM; Welch’s *t*-test *p* = 0.016 and BH FDR = 0.063. Experimental data are presented as mean ± SEM; *n* = 6 mice per group. ns, not significant; ** *p* < 0.01, *** *p* < 0.001, **** *p* < 0.0001. Abbreviations: BH FDR, Benjamini–Hochberg false discovery rate; DAPI, 4′,6-diamidino-2-phenylindole; Dex, dexamethasone; NLRP3, NLR family pyrin domain containing 3; PBS, phosphate-buffered saline; SEM, standard error of the mean; SONFH, steroid-induced osteonecrosis of the femoral head.

We then examined NLRP3, Caspase-3, and Bax in the femoral head after a glucocorticoid challenge. Four groups were compared: Piezo1^flox/+^ + PBS, Piezo1^flox/flox^ + PBS, Piezo1^flox/+^ + DEX, and Piezo1^flox/flox^ + DEX. NLRP3 fluorescence was similar between the two genotypes under PBS conditions. DEX markedly increased NLRP3 in Piezo1^flox/+^ mice, and this increase was significantly smaller in Piezo1^flox/flox^ mice (Figure 6A,D).

Caspase-3 and Bax followed the same pattern. Their baseline fluorescence intensities were comparable between genotypes, whereas DEX produced strong increases in Piezo1^flox/+^ mice that were significantly reduced by intestinal Piezo1 deletion (Figure 6B,C,E,F).

In GSE123568, the NLRP3-associated inflammatory signature was nominally higher in peripheral SONFH samples than in controls (Welch’s *t*-test, *p* = 0.016; BH FDR = 0.063; Figure 6G).

## 3. Discussion

This study connects an extra-skeletal intestinal serotonergic response with the vascular component of steroid-associated femoral-head injury. By combining pharmacological inhibition, intestinal epithelial-specific Piezo1 deletion, and BMEC experiments, our study extends the understanding of gut-derived serotonergic signaling to femoral-head microvascular injury. Most mechanistic studies of SONFH have focused on local pathological events, including endothelial dysfunction, thrombosis, lipid dysregulation, osteocyte death, and defective remodeling. Our findings suggest that intestinal endocrine signaling may also contribute to these processes, providing a rationale for investigating peripheral serotonergic signaling as a potential target for early intervention.

Type H vessels link vascular organization with the osteogenic niche, providing a plausible biological context for the concordance between vascular and trabecular phenotypes. The parallel preservation of vascular and trabecular structures following peripheral serotonin inhibition or intestinal Piezo1 deletion is consistent with this relationship, although it does not establish that vascular protection directly mediates the skeletal benefit.

PIEZO1 signaling appears to exert compartment-dependent effects on skeletal homeostasis. Our previous work implicated BMSC PIEZO1 in supporting osteogenic and angiogenic responses to elevated intraosseous pressure during early SONFH [3]. Ochiai et al. reported reduced PIEZO1-related mechanosensitivity in bone cells in glucocorticoid-induced osteoporosis and beneficial skeletal effects of PIEZO1 activation, involving Hes1-associated CaMKII/Akt signaling [21]. By contrast, intestinal epithelial PIEZO1 deletion in the present study was associated with reduced TPH1 induction and preservation of femoral-head vasculature. These apparently opposing effects may reflect differences in cellular identity, upstream stimuli, and downstream signaling. This distinction is relevant to therapeutic development, as systemic PIEZO1 inhibition may not reproduce the benefits of intestine-restricted deletion.

The increased intestinal PIEZO1 and TPH1 signals, without a detectable expansion of the CgA-positive cell population, suggest a change in the activity or expression profile of the existing intestinal epithelial compartment rather than an increase in enteroendocrine cell abundance. However, whether glucocorticoids directly influence PIEZO1 channel activity remains unresolved. The BMEC findings further suggest a relationship between serotonin exposure, endothelial dysfunction, and NLRP3- and apoptosis-related signaling. Increased NLRP3, Bax, and Caspase-3 signals accompanied impaired tube formation, while intestinal Piezo1 deletion reduced corresponding injury-related markers in vivo. Nevertheless, these observations do not establish complete inflammasome activation or identify the serotonin receptor responsible for the endothelial response.

The public transcriptomic datasets provided additional but limited support for the proposed mechanism. GSE126366 showed reduced glucocorticoid receptor-responsive markers following epithelial Nr3c1 deletion, whereas serotonergic genes showed nonsignificant downward trends. GSE214478 identified serotonin receptor and serotonin-handling transcripts in HUVECs, although these findings cannot define the receptor repertoire of BMECs. In GSE123568, the NLRP3-associated inflammatory signature was nominally increased in SONFH but did not meet the false-discovery-rate significance threshold (*p* = 0.016, FDR = 0.063). These datasets provide complementary biological context rather than direct causal evidence, and the principal interpretation rests on the pharmacological, genetic, and cellular experiments.

LPS priming represents an important consideration because it may independently influence intestinal serotonergic and inflammatory responses. In the additional four-group analysis (Figure S3), combined LPS and glucocorticoid exposure increased circulating 5-HT relative to the control and LPS-only groups, although the difference from glucocorticoid treatment alone was not statistically significant. These findings indicate that the combined exposure is associated with an enhanced serotonergic response but do not establish the independent contributions or potential interaction of LPS and glucocorticoids.

Several limitations should also be considered. The 5-HT concentrations used in BMEC experiments (20–100 μM) exceeded those measured in mouse serum. Accordingly, these experiments characterize concentration-dependent endothelial responses rather than directly reproducing physiological exposure. Moreover, serum 5-HT does not necessarily reflect the free serotonin concentration encountered by endothelial cells. The intracellular link between intestinal PIEZO1 and TPH1, the relevant endothelial serotonin receptors, and the cellular origin of injury-related signals in femoral-head tissues also require further clarification. The modest effect of pCPA on circulating corticosterone limits a strictly serotonin-specific interpretation. In addition, some experiments involved relatively small sample sizes; only male mice were studied, and necrotic tissue volume and articular cartilage integrity were not directly assessed.

Future studies should distinguish the individual and combined effects of LPS and glucocorticoids, identify the epithelial and endothelial signaling mechanisms involved, and evaluate prolonged exposure to lower serotonin concentrations. Studies involving both sexes, more comprehensive histopathological assessment, and clinical specimens will also be important for determining the broader relevance and therapeutic potential of intestinal serotonergic signaling in SONFH.

**Figure 7:**
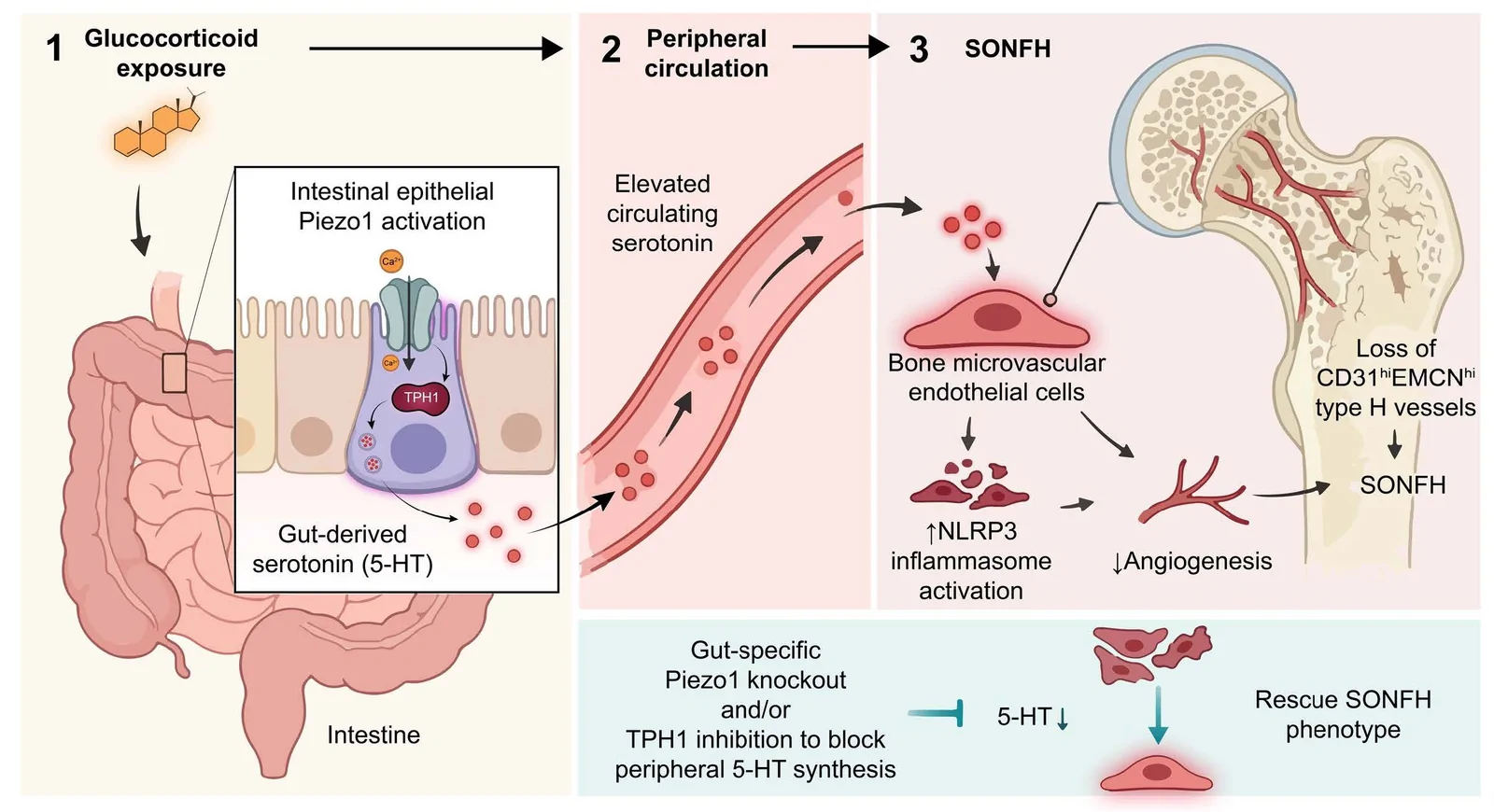
Proposed model of the intestinal Piezo1–TPH1–5-HT axis in glucocorticoid-induced SONFH. Glucocorticoid exposure increases intestinal Piezo1 and TPH1 expression together with circulating serotonin. In BMECs, serotonin increases NLRP3, Bax, and Caspase-3 and impairs tube formation. In vivo, inhibition of peripheral serotonin synthesis or intestinal Piezo1 deletion attenuates type H vessel loss and femoral-head structural injury. These findings support a pathway in which gut-derived serotonin contributes to the vascular component of SONFH. Abbreviations: 5-HT, 5-hydroxytryptamine; BMEC, bone microvascular endothelial cell; CD31, cluster of differentiation 31; EMCN, endomucin; NLRP3, NLR family pyrin domain containing 3; SONFH, steroid-induced osteonecrosis of the femoral head; TPH1, tryptophan hydroxylase 1.

Overall, the pharmacological, genetic, vascular, and BMEC findings support intestinal PIEZO1–TPH1–serotonin signaling as a contributor to femoral-head microvascular injury in the experimental SONFH model (Figure 7).

## 4. Materials and Methods

### 4.1. Study Design

We combined pharmacological inhibition of peripheral serotonin synthesis, intestinal epithelial-specific Piezo1 deletion, histological and micro-CT analyses, BMEC experiments, and public transcriptomic analysis to examine the contribution of intestinal Piezo1–TPH1–serotonin signaling to femoral-head vascular and structural injury during glucocorticoid-induced SONFH.

### 4.2. Experimental Animals

Male C57BL/6 mice aged 8–10 weeks were used in this study. Wild-type mice were obtained from the Animal Experimental Center of Tongji Medical College, Huazhong University of Science and Technology. All mice were housed under specific pathogen-free conditions with free access to food and water on a 12-h light/dark cycle at controlled temperature and humidity. All animal experiments were approved by the Animal Ethics Committee of Tongji Medical College, Huazhong University of Science and Technology, and were conducted in accordance with institutional guidelines for the care and use of laboratory animals. All personnel involved in the experiments were appropriately qualified. For pharmacological experiments, male C57BL/6 mice were used. For genetic studies, intestinal epithelial-specific Piezo1 conditional knockout mice were generated by crossing Piezo1^flox/flox^ mice with Villin-Cre transgenic mice. Offspring carrying both the floxed Piezo1 allele and Villin-Cre were designated as Villin-Piezo1^flox/flox^ mice, whereas Piezo1flox/+ littermates were used as controls. Genotyping was performed using genomic DNA isolated from tail biopsies. Mice were randomly assigned to experimental groups, and quantitative image-based and histological analyses were performed by investigators blinded to group allocation. Unless otherwise stated, in vivo experiments used *n* = 6 mice per group. Ethics approval was obtained under protocol 2706 (approved 12 March 2021), as stated in the Institutional Review Board Statement. The Villin-Cre and Piezo1 floxed breeding lines were maintained through the laboratory of Prof. Tao Bai (Division of Gastroenterology, Union Hospital, Huazhong University of Science and Technology).

### 4.3. Glucocorticoid-Induced SONFH Model and Drug Administration

A glucocorticoid-induced SONFH model was established according to a previously reported protocol [3]. Briefly, animals received intraperitoneal injections of lipopolysaccharide (LPS, 100 μg/kg) once daily for three consecutive days, followed by intramuscular injections of methylprednisolone sodium succinate (MPS, 100 mg/kg) once daily for three consecutive days. One week later, MPS was further administered at 40 mg/kg twice weekly for four additional weeks. Control animals received an equal volume of normal saline in parallel. For experiments designed to assess glucocorticoid-associated intestinal Piezo1–TPH1 activation, dexamethasone (Dex) was administered using the same LPS/glucocorticoid schedule: after LPS priming for three consecutive days, Dex was administered intramuscularly at 100 mg/kg once daily for three consecutive days, followed one week later by 40 mg/kg twice weekly for four weeks. To inhibit serotonin synthesis, p-chlorophenylalanine methyl ester (pCPA; Sigma-Aldrich, St. Louis, MO, USA, C3635) was dissolved in PBS and administered by intraperitoneal injection at 300 mg/kg once daily for five consecutive days, according to previously reported serotonin-depletion protocols [22]. Vehicle-treated mice received volume-matched PBS injections. Unless otherwise specified, pCPA treatment was initiated at the beginning of glucocorticoid induction. For the additional serum 5-HT comparison (Supplementary Figure S3), four exposure groups were compared: Control, LPS-only, GC-only, and LPS + GC.

### 4.4. Serum Corticosterone and Serotonin Measurement

At the indicated time points, whole blood samples were collected from mice and allowed to clot at room temperature. Serum was isolated by centrifugation and stored at −80 °C until further analysis. Serum corticosterone (CORT) and 5-hydroxytryptamine (5-HT) levels were measured using commercially available enzyme-linked immunosorbent assay kits according to the manufacturers’ instructions. Each sample was assayed in duplicate.

### 4.5. Tissue Collection and Preparation

At the experimental endpoint, mice were euthanized, and intestinal tissues, including the duodenum and colon, were rapidly harvested and rinsed with cold phosphate-buffered saline (PBS). Samples were either fixed in 4% paraformaldehyde for histological analysis or snap-frozen in liquid nitrogen for RNA and protein extraction. Femoral heads were carefully dissected free of soft tissues and processed for histological staining, immunofluorescence staining, or micro-computed tomography (micro-CT) analysis.

### 4.6. Hematoxylin and Eosin Staining

Femoral head specimens were fixed in 4% paraformaldehyde, decalcified, embedded in paraffin, and sectioned. Sections were stained with hematoxylin and eosin using standard procedures. Histopathological evaluation focused on trabecular integrity, marrow architecture, empty osteocyte lacunae, and osteonecrotic changes. Representative images were acquired under a light microscope by investigators blinded to group allocation. For empty osteocyte lacunae, lacunae without a visible osteocyte nucleus were counted within evaluable trabecular bone regions. The empty-lacunae proportion was calculated as the number of empty lacunae divided by all evaluable osteocyte lacunae, multiplied by 100. Individual animals rather than image fields were treated as the biological unit of analysis.

### 4.7. Immunohistochemistry Analysis

For immunohistochemical detection of TPH1, paraffin-embedded intestinal sections were deparaffinized, rehydrated, subjected to antigen retrieval, and incubated with normal serum. Sections were then incubated with anti-TPH1 primary antibody overnight at 4 °C, followed by incubation with HRP-conjugated secondary antibody. Immunoreactivity was visualized using diaminobenzidine, and nuclei were counterstained with hematoxylin. TPH1-positive cells were quantified in randomly selected microscopic fields using ImageJ software version 1.54u8.

### 4.8. Immunofluorescence Analysis

For immunofluorescence staining, paraffin-embedded tissue sections were deparaffinized, rehydrated, and subjected to antigen retrieval as required, whereas cultured cells were fixed, permeabilized, and incubated with blocking solution using standard procedures. After blocking with normal serum, samples were incubated overnight at 4 °C with primary antibodies against Piezo1, chromogranin A (CgA), CD31, endomucin (EMCN), NLRP3, Caspase-3, or Bax. After washing, sections or cells were incubated with Alexa Fluor-conjugated secondary antibodies and counterstained with DAPI. For intestinal tissues, Piezo1 and CgA double staining was performed to evaluate Piezo1 expression and enterochromaffin cell distribution. For femoral head tissues, CD31 and EMCN staining were used to identify type H vessels, whereas NLRP3, Caspase-3, and Bax staining were used to assess inflammatory activation and apoptosis-related injury. Immunofluorescence signals were visualized using a fluorescence microscope or confocal microscope, and fluorescence intensity or positive area was quantified using ImageJ software.

### 4.9. Micro-Computed Tomography Analysis

Femoral heads were harvested and fixed in 4% paraformaldehyde. Samples were scanned using a micro-CT system, and three-dimensional reconstruction was performed using the manufacturer’s software. The region of interest was defined in the trabecular bone area of the femoral head using the same anatomical boundaries across all groups. Quantitative bone morphometric parameters, including bone volume fraction (BV/TV), trabecular thickness (Tb.Th), trabecular separation (Tb.Sp), and trabecular number (Tb.N), were analyzed. Quantitative micro-CT analysis was performed with *n* = 6 femoral head specimens per group.

### 4.10. RNA Extraction and Real-Time Quantitative Polymerase Chain Reaction

Total RNA was extracted from intestinal tissues or cultured bone microvascular endothelial cells (BMECs) using TRIzol reagent according to the manufacturer’s instructions. RNA concentration and purity were measured using absorbance at 260 and 280 nm with a NanoDrop 2000 spectrophotometer (Thermo Fisher Scientific, Waltham, MA, USA). RNA was reverse-transcribed using a PrimeScript RT reagent Kit (TaKaRa Bio Inc., Kusatsu, Shiga, Japan). Gene expression was measured by real-time quantitative polymerase chain reaction using SYBR Premix (TaKaRa Bio Inc., Kusatsu, Shiga, Japan) in a Bio-Rad CFX-96 system (Bio-Rad, USA). Each reaction was performed in triplicate. Relative gene expression was calculated using the 2^−ΔΔCt^ method, and β-actin or GAPDH was used as the internal control for normalization. Unless otherwise stated, RT-qPCR analyses used *n* = 6 biological replicates per group; the Dex-validation RT-qPCR experiments shown in Figure 2E,F used *n* = 3 biological replicates per group.

### 4.11. Western Blot Analysis

Proteins were extracted from intestinal tissues or BMECs using RIPA lysis buffer containing protease inhibitors. Protein concentrations were determined using a bicinchoninic acid protein assay kit according to the manufacturer’s instructions. Equal amounts of protein were subjected to sodium dodecyl sulfate–polyacrylamide gel electrophoresis, transferred to polyvinylidene difluoride membranes, and blocked with 5% non-fat milk. Membranes were incubated with primary antibodies overnight at 4 °C, followed by incubation with HRP-conjugated secondary antibodies. Protein bands were visualized using enhanced chemiluminescence reagents and quantified by densitometric analysis using ImageJ software. Western blot quantification was based on *n* = 6 biological replicates per group unless otherwise stated; the Dex-validation blots in Figure 2G,H and the BMEC NLRP3 blot in Figure 5G used *n* = 3 biological replicates per group.

### 4.12. Bone Microvascular Endothelial Cell Culture

BMECs were cultured in endothelial cell growth medium supplemented with fetal bovine serum and antibiotics under standard conditions at 37 °C in a humidified incubator containing 5% CO_2_. Cells at appropriate passages were used for subsequent experiments. For in vitro stimulation, BMECs were treated with 0, 20, 50, or 100 μM 5-HT for the indicated duration, whereas untreated cells served as controls. In vitro experiments were performed with *n* = 6 independent biological replicates unless otherwise stated.

### 4.13. Tube Formation Assay

For in vitro tube formation assays, 96-well plates were pre-coated with Matrigel (Corning, New York, NY, USA). BMECs were seeded into the wells and incubated under the indicated conditions. Images of tube-like structures were captured after incubation, and the number of tube loops was quantified using ImageJ software. Tube formation analysis was performed with *n* = 6 independent biological replicates per group.

### 4.14. Immunofluorescence Staining of Cultured Cells

For cellular immunofluorescence analysis, BMECs were cultured on coverslips, fixed with 4% paraformaldehyde, permeabilized with Triton X-100, and blocked with normal serum. Cells were incubated with primary antibodies overnight at 4 °C, followed by incubation with Alexa Fluor-conjugated secondary antibodies. Nuclei were counterstained with DAPI. Images were acquired under identical exposure settings, and fluorescence intensity was quantified using ImageJ software. Quantification was performed with *n* = 6 biological replicates per group unless otherwise stated.

### 4.15. Public Transcriptomic Analysis

GSE126366 [23] was analyzed for GR-response and serotonergic/enteroendocrine genes after intestinal epithelial Nr3c1 deletion. GSE123568 [24] was used to score inflammatory, NLRP3-associated, apoptosis, hypoxia, endothelial-activation, and coagulation/platelet-activation signatures in peripheral SONFH samples. GSE214478 [25] provided HUVEC RNA-seq data for serotonin receptor and serotonin-handling transcript detection. Two-group comparisons used Welch’s *t*-tests, with Benjamini–Hochberg false-discovery-rate correction for signature-level analyses where applicable.

### 4.16. Statistical Analysis

Data are presented as mean ± SEM. Statistical analyses were performed using GraphPad Prism 8.0 and IBM SPSS Statistics 32.0. Comparisons between two groups were performed using unpaired Student’s *t*-tests, whereas comparisons among multiple groups were analyzed using one-way ANOVA or two-way ANOVA as appropriate. Public transcriptomic comparisons used Welch’s *t*-tests with Benjamini–Hochberg false-discovery-rate adjustment for signature-level analyses. Unless otherwise stated, in vivo experiments used *n* = 6 mice per group and in vitro experiments used *n* = 6 independent biological replicates per group. Exceptions were the Dex-validation RT-qPCR and Western blot experiments in Figure 2E–H and the BMEC NLRP3 Western blot in Figure 5G, which used *n* = 3 biological replicates per group. A *p* value < 0.05 was considered statistically significant. Distributional assumptions underlying parametric analyses were assessed using Shapiro–Wilk tests for normality and Levene’s tests for homogeneity of variance; no substantial violations were identified in the relevant comparisons.

## 5. Conclusions

Glucocorticoid exposure increased intestinal Piezo1–TPH1 signaling and circulating serotonin in parallel with femoral-head vascular and structural injury. Reducing peripheral serotonin synthesis or deleting intestinal epithelial Piezo1 mitigated these changes, while serotonin directly impaired BMEC angiogenic function and increased NLRP3, Bax, and Caspase-3. These results show that intestinal Piezo1–TPH1–serotonin signaling contributes to the vascular component of SONFH in this model.

## Data Availability

The public transcriptomic datasets analyzed in this study are available from the NCBI Gene Expression Omnibus under accession numbers GSE126366, GSE123568, and GSE214478. Other data supporting the findings of this study are available from the corresponding author upon reasonable request.

## Supplementary Materials

The following supporting information can be downloaded at https://www.mdpi.com/article/10.3390/ijms27209213/s1.

## Abbreviations

5-HT, 5-hydroxytryptamine; ANOVA, analysis of variance; APS, antiphospholipid syndrome; BH FDR, Benjamini–Hochberg false discovery rate; BMEC, bone microvascular endothelial cell; BV/TV, bone volume fraction; CD31, cluster of differentiation 31; CgA, chromogranin A; CI, confidence interval; CORT, corticosterone; DAPI, 4′,6-diamidino-2-phenylindole; Dex, dexamethasone; EMCN, endomucin; GC, glucocorticoid; GR, glucocorticoid receptor; H&E, hematoxylin and eosin; HUVEC, human umbilical vein endothelial cell; IF, immunofluorescence; IHC, immunohistochemistry; LPS, lipopolysaccharide; micro-CT, micro-computed tomography; MPS, methylprednisolone; mRNA, messenger RNA; NC, normal control; NLRP3, NLR family pyrin domain containing 3; PBS, phosphate-buffered saline; pCPA, p-chlorophenylalanine methyl ester; RNA-seq, RNA sequencing; RT-qPCR, reverse-transcription quantitative polymerase chain reaction; SEM, standard error of the mean; SONFH, steroid-induced osteonecrosis of the femoral head; Tb.N, trabecular number; Tb.Sp, trabecular separation; Tb.Th, trabecular thickness; TPH1, tryptophan hydroxylase 1; WB, Western blotting.
